# Physical Activity, Inactivity, and Sedentary Behaviors: Definitions and Implications in Occupational Health

**DOI:** 10.3389/fpubh.2018.00288

**Published:** 2018-10-05

**Authors:** David Thivel, Angelo Tremblay, Pauline M. Genin, Shirin Panahi, Daniel Rivière, Martine Duclos

**Affiliations:** ^1^Clermont Auvergne University, EA 3533, Laboratory of the Metabolic Adaptations to Exercise under Physiological and Pathological Conditions, Clermont-Ferrand, France; ^2^CRNH-Auvergne, Clermont-Ferrand, France; ^3^Département de L'éducation Physique, Faculté des Sciences de L'éducation, Université Laval, Québec City, QC, Canada; ^4^Département de Kinésiologie, Université Laval, Québec City, QC, Canada; ^5^INRA, UMR 1019, Clermont-Ferrand, France; ^6^University Clermont 1, UFR Medicine, Clermont-Ferrand, France; ^7^Département de Médecine Générale, Université Toulouse III, Toulouse, France; ^8^Department of Sport Medicine and Functional Explorations, Clermont-Ferrand University Hospital, G. Montpied Hospital, Clermont-Ferrand, France

**Keywords:** physical activity, sedentary behaviors, occupational health, inactivity, tertiary employees

## Abstract

Based on the increasing evidence linking excessive sedentary behaviors and adverse health outcomes, public health strategies have been developed and constantly improved to reduce sedentary behaviors and increase physical activity levels at all ages. Although the body of literature in this field has grown, confusion still exists regarding the correct definition for sedentary behaviors. Thus, there is a need to provide a clear definition in order to distinguish sedentary behaviors from physical activity and inactivity. This paper will briefly review the most recent and accepted definitions of these concepts and illustrate their relationships. Nowadays, since most working adults spend a high proportion of their waking hours in increasingly sedentary tasks, there will be a particular focus on the field of occupational health. Finally, simple modifications in the workplace will be suggested in order to decrease sedentary behaviors.

## Introduction

The beneficial effects of physical activity have been clearly described in the literature with recent meta-analyses providing a high level of evidence regarding its impact on overall mortality (1, 2), cardiovascular disease-related mortality (3), or cancer-relate mortality (3–5). In addition to reducing the risk of mortality, regular physical activity favors healthy growth and aging and prevents the occurrence of many chronic diseases (6). The last century has been the cradle of our societies' modernization and automation favoring the occurrence and development of sedentary opportunities and behaviors. This sedentariness has lately been described as a major mortality risk factor (7), independent of physical activity (8), and ~5.3 million of deaths are attributed to physical inactivity (9).

A worker's activity has evolved throughout the last century, clearly shifting to more sedentary occupational tasks, and this “tertiarization” results in workplaces that are of particular concern. In their research, Church and colleagues reported a decrease of about 100 calories in the daily occupation-related energy expenditure over the last 50 years in the United States, which plays a significant role in the body weight of both men and women (10). To date, while few data are available regarding employees' physical activity, their sedentary time and health-related consequences have been particularly studied. Recent research including meta-analyses have clearly underlined the negative impact of seated occupational activities on overall mortality (11, 12). According to some studies, the mortality rate is increased by 2% for every seated hour and can reach up to 8% per hour when the total consecutive time spent seated is above 8 h per day (13). These statistics are part of a large body of evidence associating occupational activities with health issues, clearly urging for appropriate worksite interventions to improve tertiary employees' health.

Our societal changes, favoring the minimization of physical effort, are particularly problematic based on the assumption that individuals possess an innate tendency to conserve energy and avoid unnecessary physical exertion. This general trend to avoid energy expenditure may explain why people do not exercise regularly despite the known negative effects of physical inactivity on health (14–16). Moreover, we are currently living in a paradoxical time where our society has become more “technophilic,” favoring strategies to avoid and/or minimize physical effort (and *per se* human motion) with more time devoted to sedentary behaviors; while on the other hand, there is a growing interest and concern for healthy lifestyles. Interestingly, new pharmacologic drugs for treating non-communicable chronic diseases are being sold with the message to move more and decrease the time spent sedentary, emphasizing the importance of an active lifestyle that cannot be replaced by any pharmacologic strategies. Both recommendations and public health strategies that promote physical activity and discourage sedentariness must rest on clear and universal definitions of these concepts to avoid any equivocal and misinterpreted messages.

The aim of this brief review is to (a) provide an update on the definitions of physical activity, inactivity and sedentariness; (b) examine their roles in occupational health; and (c) suggest simple modifications in the workplace in order to decrease sedentary behaviors.

## Definitions of physical activity and sedentary behaviors

Trained, active, inactive, and sedentary are some of the terms that have been used to describe many individuals. Misuse of these adjectives by public health communications, commercial advertisements, and scientific reports often leads to biased messages and conclusions.

For the last couple of years, researchers in the fields of physical activity and sedentary behaviors, particularly members of the Sedentary Behavior Research Network (SBRN), have worked together to clarify the definitions related to physical activity, inactivity and sedentary behaviors (Table 1 presents the main definitions) (18). In 2017, a new terminology consensus was created to highlight the differences between these concepts [see Tremblay et al. (18)]. Physical activity is defined as any body movement generated by the contraction of skeletal muscles that raises energy expenditure above resting metabolic rate, and is characterized by its modality, frequency, intensity, duration, and context of practice. In 1985, Caspersen defined exercise as a subcategory of physical activity that is planned, structured, repetitive, and that favors physical fitness maintenance or development (17). Each word in this definition of physical activity is of crucial importance to properly understand its meaning. According to the last updated definition, while resting energy expenditure corresponds to an energy expenditure of one metabolic equivalent (MET), sedentary behaviors are any waking behaviors characterized by an energy expenditure ≤ 1.5 METs, while in a sitting, reclining, or lying posture (18). This last definition is of particular importance since in 2015 Gibbs and colleagues called for a better definition of sedentary behaviors that considers both intensity and posture (19). Screen time and sitting time are usually the two main indicators used to quantify the time devoted to sedentary behaviors. From an energetic and biological point of view, there is also a clear need to consider the exact nature of each sedentary behavior that may not have similar physiological consequences. Indeed, sedentary activities demanding cognitive effort favor an increase in cortisol concentrations, glycemic instability, energy intake as well as a decrease in the parasympathetic/sympathetic balance (20). Such physiological implications have to be considered since sedentary behaviors involving cognitive tasks (mental work) have the profile of an activity with very low movement and with a component of neurogenic stress (20–22).

**Table 1:** Main definitions.

**Terms**	**Definitions**
Physical activity	Any body movement generated by the contraction of skeletal muscles that raises energy expenditure above resting metabolic rate. It is characterized by its modality, frequency, intensity, duration, and context of practice (17)
Physical inactivity	Represents the non-achievement of physical activity guidelines
Exercise	Subcategory of physical activity that is planned, structured, repetitive, and that favors physical fitness maintenance or development (17)
Sport	Sport is part of the physical activity spectrum and corresponds to any institutionalized and organized practice, reined over specific rules
Sedentary behaviors	Any waking behaviors characterized by an energy expenditure ≤ 1.5 METs, while in a sitting, reclining, or lying posture (18)

Physical activity and sedentary behaviors are not the opposite of each other. Individuals are considered to be active when they reach physical activity recommendations for their age, which does not prevent them from also devoting a significant part of their time to sedentary behaviors. In other words, individuals can be classified as both active and sedentary. Tertiary employees are the most demonstrative example of sedentariness as they spend a considerable part of their time seated in front of a computer screen. This defines them as highly sedentary, while they may or may not reach their aged-related physical activity recommendations outside of work (23). This confusion mainly rests on the challenge of differentiating between sedentariness and physical inactivity that must be defined as not following physical activity guidelines. This inappropriate understanding of these terms can be illustrated using the recent paper by Rantalainen et al. (24), who compared the amount and patterns of time devoted to moderate-to-vigorous physical activities (MVPA) between two groups of habitual recreational runners: (i) running between of 20 to 40 km per week and (ii) running more than 50 km per week) and a “sedentary group” composed of office workers (24). However, to be included in their “sedentary group,” participants had to engage in < 150 min per week of MVPA, defining them as inactive and not sedentary. The misuse of the concept in this study was justified by the fact that the three groups showed a statistically similar overall sedentary time. This study clearly highlights that individuals may be classified as both active and sedentary and that inactive and sedentary must not be confused in order to avoid any misinterpretations, incorrect conclusions and/or public health messages (25).

Similarly, the term physical activity is commonly confused with sport (not at the scientific level). Sport is part of the physical activity spectrum and corresponds to any institutionalized and organized practice, based on specific rules. Some very active individuals might not be sport athletes even though they regularly train and show a high level of physical activity. This distinction is of importance with respect to public health messages since individuals and patients may fear the term “sport” while what is really required is a higher or regular amount of physical activity participation.

Once clearly understood, the adoption of these different concepts rests on individuals' behaviors. It is important to consider the behavioral dimensions of physical activity and sedentariness. To be physically active and avoid too much sedentary time is, today, a voluntary behavior. External and societal influences are strong, but these constraints must be changed into habits. Physical activity must be included as a core element of human nature and we must go back from what Epstein referred to in the nineties as “sedentary alternatives” (26) to daily “active alternatives” (27).

## Sedentary behaviors and physical activity: implications in occupational health

While the worksite has been recently suggested as a new strategic opportunity to promote physical activity, due to the important amount of time employees spend at work, the “tertiarization” of work also highlights the urgent need to fight sedentary behaviors and sedentary time during working hours. The prevalence of sedentary professions increased by 20% in the United States between 1960 and 2008, with a concomitant decline of more “physically active professions” (10). In France, working adults have been shown to spend about 10 h per day sitting on workdays (with at least 4.17 h/day seated at work) and 7.58 h/day sitting on non-workdays, with a clear association between the time spent sedentary at work and sedentary behaviors outside of work (28). These data underline that physical activity programs and interventions must be proposed to tertiary employees to increase their activity levels. Strategies that aim at breaking this sedentary time must also be conducted. Recent data have shown that among office workers, who spend at least 7 h per day seated at their desk mostly in front of a screen, health indicators such as waist circumference, body mass index, or fat mass are not improved among active employees compared to inactive ones, suggesting the potential negative impact of sedentary time over physical activity levels (29). These results are of particular importance and are associated with recommendations formulated by Rosenberg et al. calling for interventions targeting high-risk populations, such as tertiary employees (30).

Standing work stations have been proposed to break this sedentary time. Although standing stations remain inactive, according to the framework proposed by the Sedentary Behavior Research Group, passive standing corresponds to 2 METs, which is above the 1.5 METs threshold used to define sedentary behaviors, considered as low physical activity (18). Although this energetic cost of passive standing rests on a strong body of scientific evidence, a recent study showed that passive standing does not significantly increase heart rate and energy expenditure above rest (31). According to the authors, the observed rises in heart rate and energy expenditure are due to the transition from sitting to standing before returning to resting values, particularly in “energy saver individuals” (31). This may explain why some studies failed to find any effects of standing desk allocation vs. classical sitting on metabolic profiles and body composition among tertiary employees (32). This could also explain why regular sitting breaks have been shown to improve health compared with permanent passive standing positions (33). In their research, Bailey and Locke showed that only active breaks consisting in brief bouts of light-intensity activities (2-min walk every 20 min) but not passive standing breaks might enhance cardiometabolic health in tertiary employees (34). Although further research is needed regarding the exact effects of standing desks and regular breaks, active standing (18) such as walking and cycling desks or walking breaks should be prescribed, regardless of the employees' physical activity level. Even though new investigations are warranted, some promising results already demonstrate the beneficial effect of walking or bike-desks on overall health, well-being, and work-related cognitive performance among tertiary workers (35). Some recent findings have also underlined the cardiometabolic benefits obtained by interrupting sitting time by the use of active walking desks compared with prolonged sitting (36). Future research should consider a potential inter-individual variability in the responses to such strategies, with some people that might adopt compensatory mechanisms leading to increased sedentary time outside of work, for instance.

While worksites have been pointed out as new ideal settings to promote physical activity, the complexity of tertiary activities that by definition favor sedentariness, combined with the independent effect of sedentary time and physical activity on health, must lead stakeholders and practitioners to conduct individualized interventions not only favoring physical activity but also, breaking this sedentary time. Even though it has been suggested that performing 60–70 min of moderate physical activity per day could eliminate the deleterious impact of sitting time, it does not eliminate the increased risk associated with screen time (37). Moreover, only a small proportion of the population reaches such an amount of daily physical activity, which must reinforce individualized strategies. It is important to note that breaking apart sedentary times and having little bouts of light physical activity is the beginning of human mobility for our tertiary physically inactive and sedentary bodies, whose genes were programmed 40,000 years ago to walk not only 30 min a day (2.5 km) but 20 km per day (38).

## Author contributions

All authors listed have made a substantial, direct and intellectual contribution to the work, and approved it for publication.

### Conflict of interest statement

The authors declare that the research was conducted in the absence of any commercial or financial relationships that could be construed as a potential conflict of interest.
